# A narrative commentary about interoperability in medical devices and data used in diabetes therapy from an academic EU/UK/US perspective

**DOI:** 10.1007/s00125-023-06049-5

**Published:** 2023-12-02

**Authors:** Johan Jendle, Peter Adolfsson, Pratik Choudhary, Klemen Dovc, Alexander Fleming, David C. Klonoff, Julia K. Mader, Nick Oliver, Jennifer L. Sherr, Jan Šoupal, Lutz Heinemann

**Affiliations:** 1 School of Medicine, Institute of Medical Sciences, Örebro University, Örebro, Sweden; 2 Department of Paediatrics, The Hospital of Halland Kungsbacka, Kungsbacka, Sweden; 3 Institute of Clinical Sciences, Sahlgrenska Academy at University of Gothenburg, Gothenburg, Sweden; 4 Diabetes Research Centre, University of Leicester, Leicester, UK; 5 Faculty of Medicine, University of Ljubljana, Ljubljana, Slovenia; 6 Department of Paediatric Endocrinology, Diabetes and Metabolic Diseases, University Children’s Hospital, Ljubljana, Slovenia; 7 Kinexum, Harpers Ferry, WV USA; 8 Diabetes Research Institute, Mills-Peninsula Medical Center, San Mateo, CA USA; 9 Division of Endocrinology and Diabetology, Department of Internal Medicine, Medical University of Graz, Graz, Austria; 10 Department of Metabolism, Digestion and Reproduction, Faculty of Medicine, Imperial College London, London, UK; 11 Yale University School of Medicine, New Haven, CT USA; 12 3rd Department of Internal Medicine, 1st Faculty of Medicine, Charles University, Prague, Czech Republic; 13 Science Consulting in Diabetes GmbH, Düsseldorf, Germany

**Keywords:** Big data, Diabetes therapy, Glucose monitoring, Insulin delivery systems, Interoperability, Medical devices, Review

## Abstract

People living with diabetes have many medical devices available to assist with disease management. A critical aspect that must be considered is how systems for continuous glucose monitoring and insulin pumps communicate with each other and how the data generated by these devices can be downloaded, integrated, presented and used. Not only is interoperability associated with practical challenges, but also devices must adhere to all aspects of regulatory and legal frameworks. Key issues around interoperability in terms of data ownership, privacy and the limitations of interoperability include where the responsibility/liability for device and data interoperability lies and the need for standard data-sharing protocols to allow the seamless integration of data from different sources. There is a need for standardised protocols for the open and transparent handling of data and secure integration of data into electronic health records. Here, we discuss the current status of interoperability in medical devices and data used in diabetes therapy, as well as regulatory and legal issues surrounding both device and data interoperability, focusing on Europe (including the UK) and the USA. We also discuss a potential future landscape in which a clear and transparent framework for interoperability and data handling also fulfils the needs of people living with diabetes and healthcare professionals.

**Graphical Abstract:**

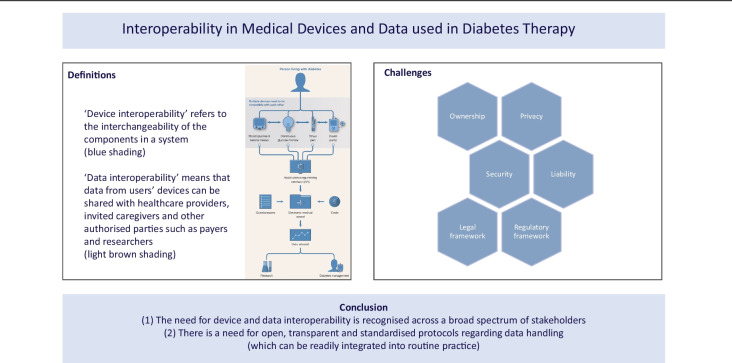

**Supplementary Information:**

The online version contains a slideset of the figures for download available at 10.1007/s00125-023-06049-5.

## Introduction

**Fig. 1:**
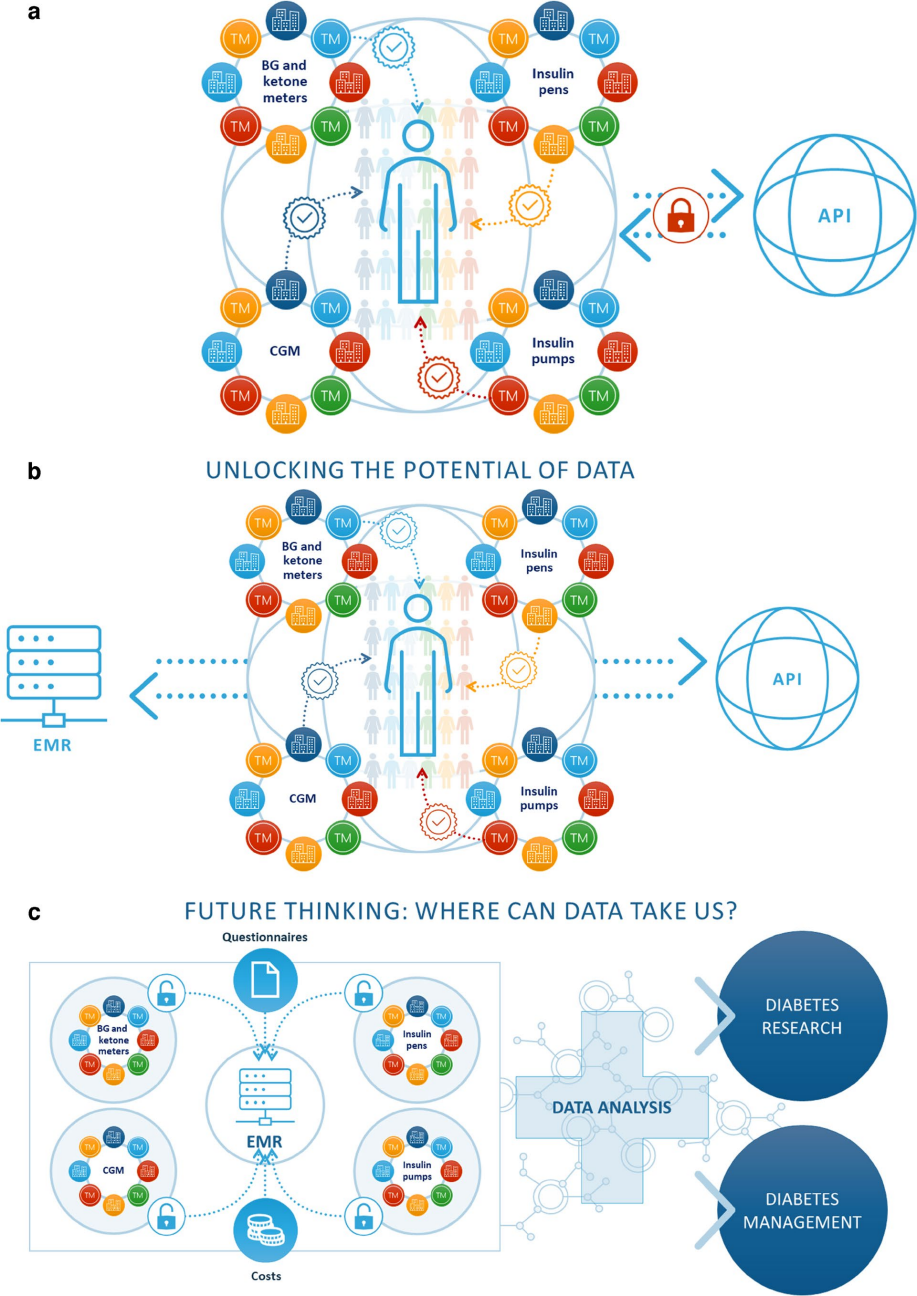
Device and data interoperability and the way ahead. Regarding the availability of medical devices for diabetes therapy, across most developed markets there are currently at least ten different continuous glucose monitor (CGM) systems available, at least 15 different insulin pumps, at least ten connected insulin pens or cap systems (also known as ‘smart pens’ [29, 30] and at least 150 systems for the measurement of (capillary) blood glucose (BG) and ketone body levels. Systems for the continuous monitoring of ketones will also enter the market soon [31]. (**a**) In this scenario, an API, which would allow the integration of data from different devices into a site’s/organisation’s EMR system, is not available, represented by the red padlock. As different brands are not always compatible with each other or with diabetes management software, fully personalised treatment is hampered. (**b**) Unlocking the potential of data. In this scenario, an open API, illustrated by removal of the red padlock, creates opportunities to use data from different devices for additional purposes, including data sharing and data documentation. (**c**) Future thinking: where can data take us? In this scenario the potential of data is unlocked, that is, the red padlocks are removed and there is free data flow between the devices and the EMR. These data also enable the costs of different treatment procedures to be calculated. Additional information from patient questionnaires and HCPs can be transferred to the EMR. The combined information allows advanced data analysis and further diabetes research and optimisation of diabetes management. Different devices (‘TM’) and companies (images of buildings) are represented in each group (BG and ketone meters, CGM, insulin pens and insulin pumps) in different-coloured circles. The PLWD is illustrated in the centre of the figures and individual patient choices are illustrated by the different-coloured ticks. TM, trademark. Images (**a**), (**b**) and (**c**) are provided courtesy of Novo Nordisk. These images were initially presented by Adolfsson P. at ATTD 2022, Barcelona [32]. Used with permission. This figure is available as part of a downloadable slideset

**Fig. 2:**
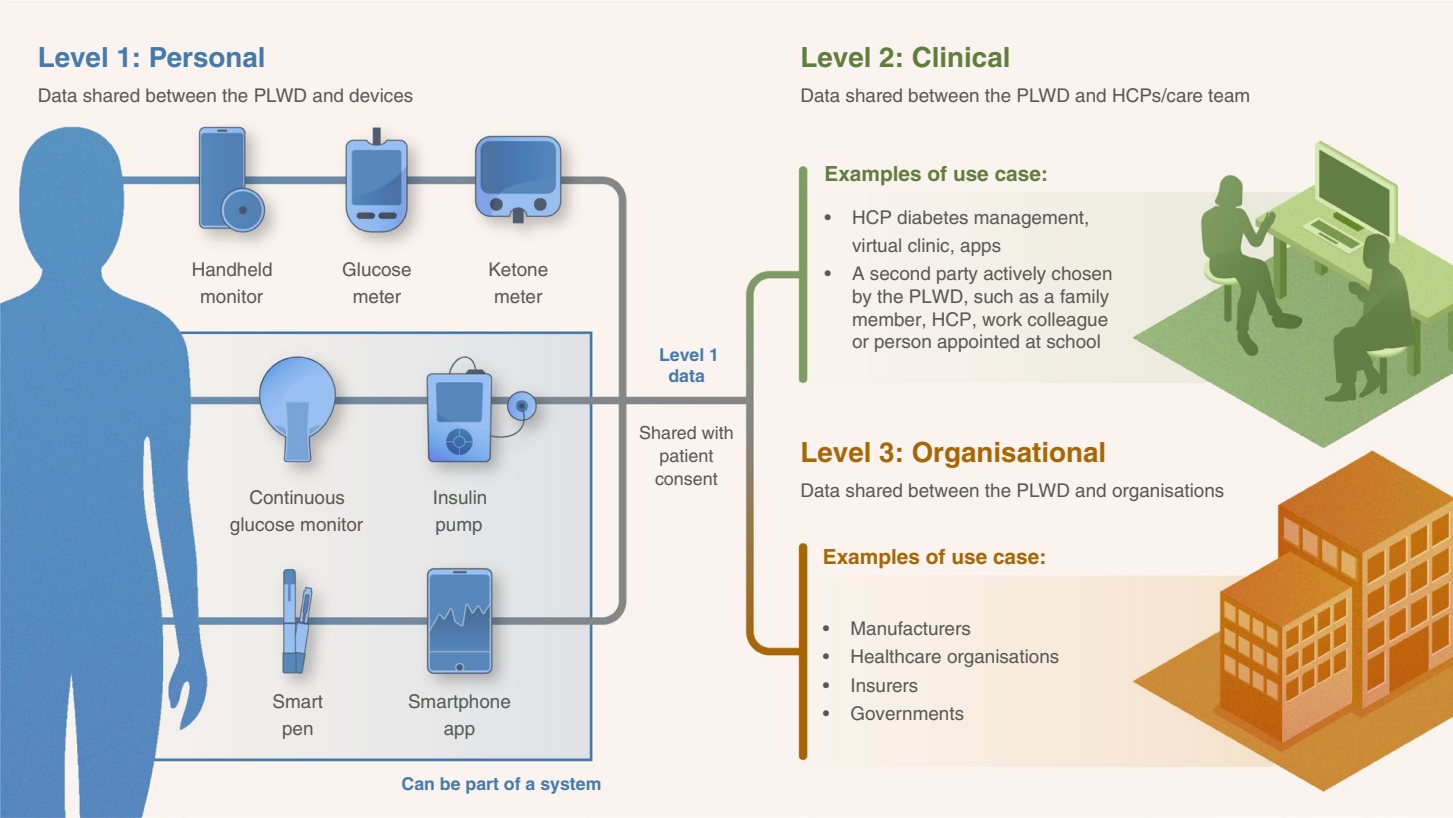
Present diabetes management and the handling of data from devices and access to data/device ecosystems can be broadly divided into three levels that require different degrees of data interoperability. At level 1, data are shared between the PLWD and the devices. At level 2, the data are shared between the PLWD and their HCPs/care team. At level 3, the data are shared between the PLWD and organisations (manufacturers, healthcare organisations, insurers and governments). Image designed by Science Graphic Design (UK). Used with permission. This figure is available as part of a downloadable slideset

Many different medical devices are available to support people living with diabetes (PLWD) with diabetes management. However, to get the most benefit from these devices it is important that they communicate effectively with each other (device interoperability) and that the data generated are seamlessly integrated as well as viewed across platforms (data interoperability) (Figs 1 and 2). Data are invaluable for PLWD and their healthcare professionals (HCPs); however, issues related to data generation, transfer, storage, access and use must be considered. Several factors currently limit the full potential of the data generated by these medical products. Some are hurdles regarding costs and regulatory policies; however, many are related to data sharing. One major question in this respect is related to data ownership, which might look different in different countries. HCPs and, to a greater extent, device users are end-users and the data generated are crucial to population research and large-scale real-world evidence (RWE) studies.

A key objective in enabling data interoperability is to have a standard application programming interface (API), which would allow the integration of data from different devices into a site’s/organisation’s electronic medical record (EMR) system, with a single point of entry for the system [1] (Fig. 1). An open API enables software developers to handle data from multiple sources, for example for use in virtual diabetes clinics (VDCs). However, a VDC could require multiple data platforms and multiple sign-ins, which would be time-consuming and hinder efficient workflows. Consequently, a simplified process enabling the use and integration of data transfer from different products into digital platforms would reduce the burden for both PLWD and their HCPs, with automated or semi-automated data transfer making the process smoother [1]. The seamless transmission and interpretation of data could also be made more accessible through agreement on a minimum dataset and standardisation of interoperability between medical devices, device-specific software, EMR systems, apps and HCP interface portals [2]. Open access to data, while complying with the General Data Protection Regulation (GDPR), which came into effect in the EU in 2018, would also support data analysis, data interpretation and display of data in a clear and instructive manner [3]. Ongoing initiatives such as the integration of Continuous Glucose Monitoring Data into the Electronic Health Record (iCoDE) project are currently focusing on providing a data standard and workflow framework for integrating continuous glucose monitoring (CGM) data into EMRs to enable these data to be readily accessed and used in routine clinical care [4]. iCoDE is based on the US Health Insurance Portability and Accountability Act of 1996. A European version of iCoDE would need to be compliant with the GDPR. In addition to use by HCPs, manufacturers also use device-specific data for proprietary data management systems and quality control, as required by post-marketing surveillance requirements set out in the 2017 EU Medical Device Regulation (MDR) [5], wherein the manufacturer is responsible for the information presented by the proprietary data management system (not for third-party interpretation).

This article provides an overview of critical issues around interoperability in medical devices and data used in diabetes therapy, including data ownership, privacy, security, interoperability limitations and the influence of interoperability on the device marketplace. It also discusses the main challenges that must be addressed for device and data interoperability to generate a seamless, secure process that complies with local regulatory and legal requirements in Europe (including the UK) and the USA. For definitions related to device and data operability, see the textbox (‘Device and data interoperability: definitions’). This article is not intended to be a systematic (scientific) review or a consensus paper about interoperability. In a sense, it is more an academic commentary, mainly from an EU/UK/US perspective. We are fully aware of the fact that interoperability is a complex topic that undergoes rapid changes. One limitation of our approach is that we are a group of academics with a focus on the clinical point of view; consideration from other stakeholders is also needed. 
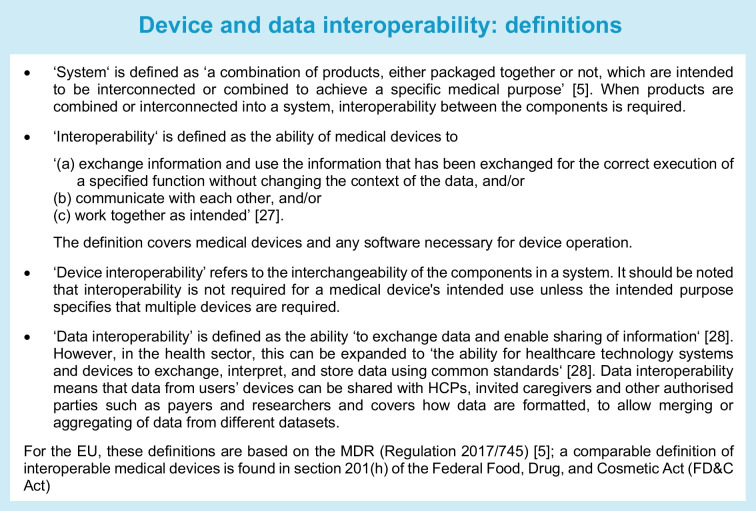


## Limitations of interoperability

Device interoperability is particularly vulnerable to limitations such as cybersecurity, quality and liability in terms of a system’s intended purpose. While the intended purpose of CGM systems is glucose monitoring and that of insulin pumps is insulin delivery, the intended use of automated insulin delivery (AID) systems is diabetes management as driven by an AID algorithm, the accuracy of insulin delivery and input by the CGM system. In the USA, the US Food and Drug Administration (FDA) tends to regulate end-to-end systems but differences in sensor accuracy, algorithms or insulin delivery, in particular, may impact clinical outcomes. On a similar note, the efficacy and safety of a system’s performance is based on the engineering specifications of the individual components and not on the performance in general of the system.

From the user perspective, using a combination of different products means that there may be some gaps in customer care that arise from interoperability considerations, as well as restricted or piecemeal clinical and technical support for HCPs. Additionally, the use of different products means that there is the potential for discrepancies within a model of open data handling with different devices from different manufacturers. For example, a user may have indicated to the manufacturer of a given CGM system that a third party may use their data. However, the user may not have granted permission for the pump manufacturer to use their data or may initially grant permission to both manufacturers and subsequently withdraw permission from one manufacturer but not the other. In this situation, it is necessary to consider how the CGM information may be passed from one manufacturer to the other. Another potential legal concern around combined products is that, in the eventuality of a malfunction, who is liable and how is this determined?

Given the limited computational power of AID systems, the checking of data integrity, imputation of missing data and reconciliation of outliers in data by these systems are limited. Hence, the coordination of such activities by various devices becomes another challenge for the interoperability of medical devices. Potential limitations from the regulatory perspective concerning AID system approval include that some markets have defined diabetes-specific operability standards but have not yet created the approval process for the system in its intended use. At the same time, new AID technologies are bringing new concepts regarding a ‘hands-off’ approach to glucose control. Therefore, it is likely that at least some of the challenges for users and HCPs in terms of coping with the volume of data and the provision of therapy recommendations by HCPs will be overcome by design.

## Regulatory and legal frameworks

### Overview

There are substantial differences across jurisdictions concerning the regulatory and legal frameworks for medical products and devices. The regulation of medical devices in the USA (where the FDA is the single national agency responsible for device approval) and the EU (where different notified bodies are designated and contracted for device review and approval) differ considerably in terms of requirements and organisational structure. There are differences across jurisdictions in terms of how data/RWE can be used. Companies may be subject to specific laws depending on where the company headquarters are located, where the diabetes technology devices are manufactured and where the servers for data storage are located. For example, the legal frameworks for data protection differ between Europe and the USA [6, 7].

### Regulatory frameworks in the EU/UK/USA

The EU does not have an interoperable diabetes device pathway comparable to that in the USA. Instead, in the EU, the manufacturer specifies the intended purpose, technical specifications, indications and limits of use for its product supported by clinical evidence, which are detailed in the instructions/information for users and the technical documentation. Within the EU, the European Medicines Agency (EMA) is responsible for the regulation of some medicines but not for the regulation of medical devices; instead, the competent authorities are the regulators of medical devices and accredit notified bodies in Europe to assess manufacturers [8].

Since May 2017, a new regulatory framework for medical devices (MDR) has been in place in the EU [5], which represents a significant change in how they are regulated [9]. The implementation of the MDR commenced in May 2021 and, from May 2024, all medical devices placed on the market will have to conform to the MDR. Medical devices are evaluated and those that conform to the EU MDR are allowed to enter the market by the independent notified bodies. The MDR requires the manufacturer to provide technical documentation and perform clinical evaluation. Traditionally, medical devices, but not necessarily diabetes-related products, have reached the market sooner in the EU than in the USA. However, implementation of the MDR may reduce the differences in data requirements and marketing approval times between the EU and the USA. In particular, the MDR has new, stricter classification rules for AID systems (e.g. Rule 22, ‘Active therapeutic devices with an integrated or incorporated diagnostic function that significantly determines the patient management’) with a higher risk classification (class III) and is more complex than the modular US approach. In addition, unlike the USA, there are no standard technical specifications for any of the different devices within AID systems. Furthermore, no clear direction or guidance exists on how notified bodies should practically apply the definition of AID systems and the higher risk classification through their reviews and issuance of certificates.

### Combining products from different manufacturers

The manufacturer of a system component defines its interoperability with other components. In the area of diabetes, for example, this results in the availability of AID system components intended to be combined only with other specified system components (e.g. from the same manufacturer), as well as the availability of system components intended to be used with a wider range of components (e.g. from different manufacturers). In instances where components from different manufacturers are combined, interoperability must be explicitly demonstrated (i.e. similar to the FDA’s interoperability provision). Key considerations for combined products include establishing who is responsible for the combined product in terms of liability and warranty and how to demonstrate the safety and efficacy of combined devices in accordance with the MDR. With regard to AID systems, other components might be added for continuous monitoring of physical activity or ketone bodies.

### Liability in Europe

According to the EU Product Liability Directive, the manufacturer of a device is liable for the device and should ensure that it is working according to the product’s specifications, as formalised in the CE (Conformité Européenne) mark and the instructions for use (IFU) (per EU Council Directive 85/374/EEC of 1985 on the approximation of the laws, regulations and administrative provisions of the Member States concerning liability for defective products [10]. The labelling or IFU is defined in the MDR as the ‘information provided by the manufacturer to inform the user of a device’s intended purpose and proper use, and of any precautions to be taken’, and the manufacturer is defined as the ‘natural or legal person who manufactures or fully refurbishes a device or has a device designed, manufactured or fully refurbished, and markets that device under its name or trademark’ [5]. However, in terms of liability, the PLWD who uses the device also has a degree of responsibility in that they are expected to use the device according to the IFU provided by the manufacturer. Therefore, these instructions need to be clear, transparent and understandable. Some manufacturers provide specific warnings to intended users in case of misuse or modifications of a device, such as ‘modifying the devices can cause serious injury, interfere with your ability to operate the device and void your warranty’ [11, 12].

The EU’s position on interoperability should also be considered in the context of digital health, wherein the EU acknowledges the need for digital tools to support PLWD-centred care. However, many discussions about digital health and future directions are also ongoing within the EU, and the use of any digital tools should also consider the current data privacy protection guidelines (GDPR) and the corresponding data privacy provisions of the UK Data Protection Act 2018, which is the UK’s version of the GDPR [13]. Any exchange of information from PLWD will require a European EMR to be established, which in turn will require data interoperability specifications to be met, and only when data can be assessed in a standardised manner can the data generated by diabetes technology systems be successfully integrated into EMRs. However, a key component of the GDPR is the ‘right to be forgotten’ [14], which raises the questions of what happens to an individual’s data if that individual wishes to stop using a given AID system and whether this information can be appropriately deleted. Data are supposed to be stored on servers located in the EU only. From our point of view, all data generated in the context of this therapy are owned by the individual PLWD and not the manufacturer of a given device. PLWD should therefore have access to the data stored by the manufacturer and the opportunity to have these data deleted.

In Europe, several legislative initiatives have been designed to both ease and regulate access to data; these include the Artificial Intelligence Act [15], the Data Act [16] and the creation of the European Health Data Space [17]. The European Health Data Space, specifically, once formally adopted, will provide a health-specific ecosystem comprising rules, common standards and infrastructures. It will also include a governance framework that aims to empower individuals through increased digital access to and control of their electronic personal health data. This approach can work at both a national level and an EU-wide level and will support the free movement of data. Moreover, it will also foster a genuine single market for EMR systems, relevant medical devices and high-risk artificial intelligence systems. Finally, the availability of information in all EU languages, in accordance with EU regulations, will enable data searching by all individuals across the EU, providing a consistent, trustworthy and efficient set-up for using health data for research, innovation, policymaking and regulatory activities.

### Regulatory framework on interoperability in the USA

The FDA has been highly supportive of the development of diabetes technology devices, beginning with detailed guidance introduced in 2012 [18]. This guidance covered CGM systems, primary endpoints that can be used to determine safety and effectiveness, indications for use, and clinical study progression and design. Moreover, the 2012 guidance explicitly notes that the intention is to apply the ‘least burdensome’ approach to investigating and developing AID systems and making them available to PLWD. The FDA has also encouraged interoperability among available insulin pumps, CGM systems and other diabetes devices, with the specification in 2018 of an integrated CGM (iCGM) regulatory pathway for CGM systems [19]. In 2019 the FDA authorised the first interoperable insulin pump, also known as an alternate controller-enabled (ACE) insulin pump, which would allow PLWD to customise treatment through their diabetes management devices. This designation meant that an ACE insulin pump could be used with different components that make up diabetes therapy systems [20]. In 2023 the FDA approved the first interoperable automated glycaemic controller app [21]. Connected diabetes devices allow PLWD to tailor their diabetes management according to their preferences for devices.

Additionally, the FDA is also able to grant clearance for different device enhancements and new products, which is designed to ease the burden on clinical practices and allow for greater PLWD-centred care and self-management. Such features can also help PLWD to make better decisions about how and when to treat their diabetes, reduce some of the challenges associated with glucose management and reduce the burden of living with diabetes.

## Cybersecurity

In 2022, the Institute of Electrical and Electronics Engineers (IEEE) Standards Association in the USA completed IEEE 2621, a standard for wireless diabetes device security assurance. This is the first and only medical device cybersecurity standard that comprehensively evaluates performance claims by manufacturers. It is focused on wireless diabetes devices and supports interoperability. This standard includes a certification programme built with industry consensus to ensure that IEEE-certified products continuously demonstrate conformance to the standard [22]. The standard was officially recognised by the FDA in 2022 [23]. No such standards exist in the EU.

## Data ownership, privacy and security

Even though HCPs often use the data generated by medical devices and, while these are invaluable in terms of generating RWE, it is important to remember that the data are owned by the PLWD, and it is the PLWD who ultimately determines the extent to which the data are shared and can be used. That is, it is the user who consents to whether the data can be used for personal health management, research, product development or marketing. In some cases, and from a legal perspective, some may argue that questions from companies regarding data use are not straightforward, nor are the potential usages of data asked about separately. Some AID devices send data directly via a Subscriber Identification Module (SIM) card without the PLWD doing anything to the upload device; there is one AID system on the market that requires the user to be notified. To ensure essential understanding, data use questions should explicitly state the purpose and potential area of data usage. Furthermore, PLWD should be allowed to respond 'yes' or 'no' to each question. Currently, companies may ask for data for research purposes; however, no ethical approval is needed for such data use.

For data emanating from single or combined devices, security, privacy and data protection are of fundamental importance, particularly with the trend among manufacturers to use the data in RWE studies. The use of RWE is gaining increasing recognition in both clinical and political decision-making (by policymakers, regulators and payers), given that RWE studies often involve large datasets and include data from all people who have consented for their data to be used, which usually represents a more diverse cohort than those who meet inclusion criteria for clinical trials [24]. The advantage of RWE studies is that PLWD who are ineligible for clinical trials because of the inclusion/exclusion criteria (age, diagnosis of dementia, HbA_1c_ cut-off values, prone to hypoglycaemia, presence of comorbidities) are included in such datasets. Such studies therefore provide valuable insight into the effectiveness of devices in a real-world environment. However, a key question regarding RWE relates to whether device users know how their data will be used. Users are likely to indicate a willingness to donate their data for research purposes, but it should be noted that the consent should be accompanied by information on data usage. Ethics committee approvals for large datasets that ensure privacy and anonymity are not commonly required for such studies.

Other concerns regarding data privacy relate to the potential use of data by entities such as insurance companies or courts of law. For example, if data from individuals were accessible to insurance companies, would these companies be able to modify coverage? Additionally, could users refuse to share their data with HCPs if CGM data are identifiable? What would be the implications of such a refusal to share data?

## Impact of device interoperability on the device marketplace

Consumer and market forces influence interoperability; for example, a key incentive for component interoperability is the user’s ability to select specific components based on merit or reviews/opinions expressed online or on social media. Similar to other industries, the user may choose between different systems, supporting the need for different components to be available. While AID systems are considered medical devices first and foremost, they are also devices that the user has to interact with frequently during the day. In this way, AID systems may also be considered consumer devices. Therefore, providing users with a choice of components that best suits their lifestyles and wishes may help with ongoing engagement and use of the system to achieve therapeutic goals. This choice can be considered analogous to assembling a desktop computer with a choice of components compared with buying a fully assembled desktop. PLWD may want to choose a pump, an algorithm and a CGM based on their preferences, or a fully assembled system. It should be noted that neither option has been shown to be better than the other.

Interoperability between the different components needed to establish an AID system is a key hurdle for individuals who build their own systems. Such open-source AID systems are not addressed in detail here; however, their existence is in part due to the requirement for interoperability from PLWD. In the last few years a number of articles have been published that support some of the fundamental considerations presented here [25, 26].

## Summary and conclusions

Transparent guidelines/standards developed using a stepwise approach should help ensure that innovations can be approved while also accounting for data ownership, safety of the devices used, and data safety and privacy. An open and transparent standard for data handling remains to be established, with the solution needing to fulfil the requirements of the reality of routine clinical practice. Only when data can be assessed in a standardised manner can the data generated by AID systems be integrated into EMRs. There is a need for an international committee on worldwide interoperability regulations; this role could be performed by an initiative of the WHO/IDF.

Through the combined efforts of many stakeholders (including legislative, regulatory and clinical stakeholders and medical device manufacturers), considerable progress has been made in formulating practical solutions for data and device interoperability in medical devices and data used in diabetes therapy. However, there is a need for professional diabetes associations and those directly involved in the care of PLWD to improve their understanding of the requirement to consent to analysis of their data. Furthermore, it is also important to make PLWD aware of how their data may be used with different degrees of anonymity and security, for example by hospitals, insurance companies and law enforcement agencies.

Finally, given the rapid pace of development of technology used in the management of PLWD, a regular update on data and device interoperability appears advisable.

### Supplementary Information

Below is the link to the electronic supplementary material.

Supplementary file1 (PPTX 722 KB)

## Abbreviations

ACE: Alternate controller-enabled
AID: Automated insulin delivery
API: Application programming interface
CGM: Continuous glucose monitoring
EMR: Electronic medical record
EMA: European Medicines Agency
FDA: US Food and Drug Administration
GDPR: General Data Protection Regulation
HCP: Healthcare professional
iCGM: Integrated continuous glucose monitoring
iCoDE: Integration of Continuous Glucose Monitoring Data into the Electronic Health Record
IEEE: Institute of Electrical and Electronics Engineers
IFU: Instructions for use
MDR: Medical Device Regulation
PLWD: People living with diabetes
RWE: Real-world evidence
VDC: Virtual diabetes clinic
