# Supplementary material for: A narrative commentary about interoperability in medical devices and data used in diabetes therapy from an academic EU/UK/US perspective
Source: Diabetologia. 2023 Dec 2;67(2):236–45. doi: 10.1007/s00125-023-06049-5 (PMC10789828; doi:10.1007/s00125-023-06049-5)
Supplement: Supplementary file 1 — Supplementary file1 (PPTX 722 KB) [file 125_2023_6049_MOESM1_ESM.pptx]

## Slide 1
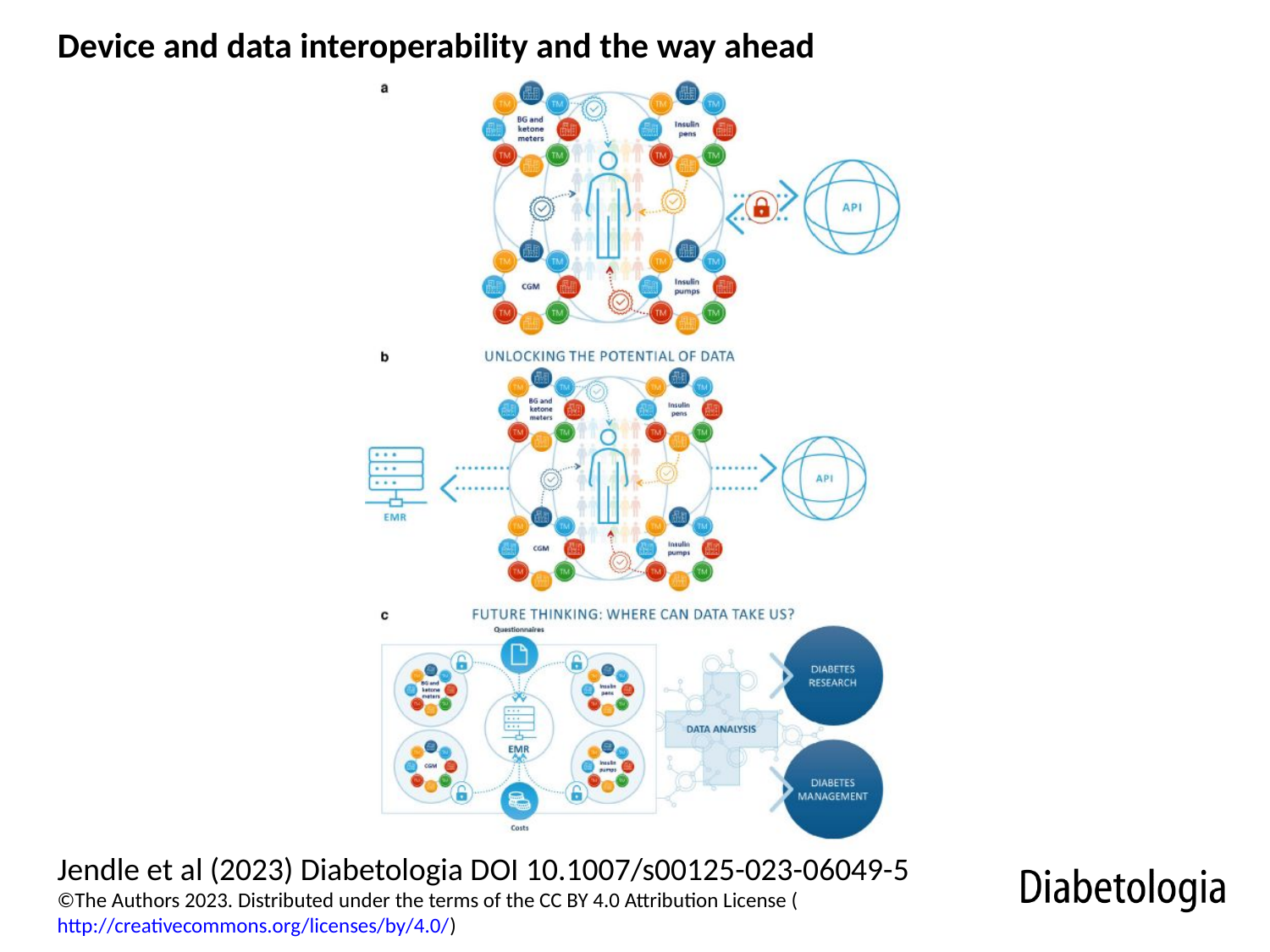

Device and data interoperability and the way ahead
Jendle et al (2023) Diabetologia DOI 10.1007/s00125-023-06049-5
©The Authors 2023. Distributed under the terms of the CC BY 4.0 Attribution License (http://creativecommons.org/licenses/by/4.0/)

## Slide 2
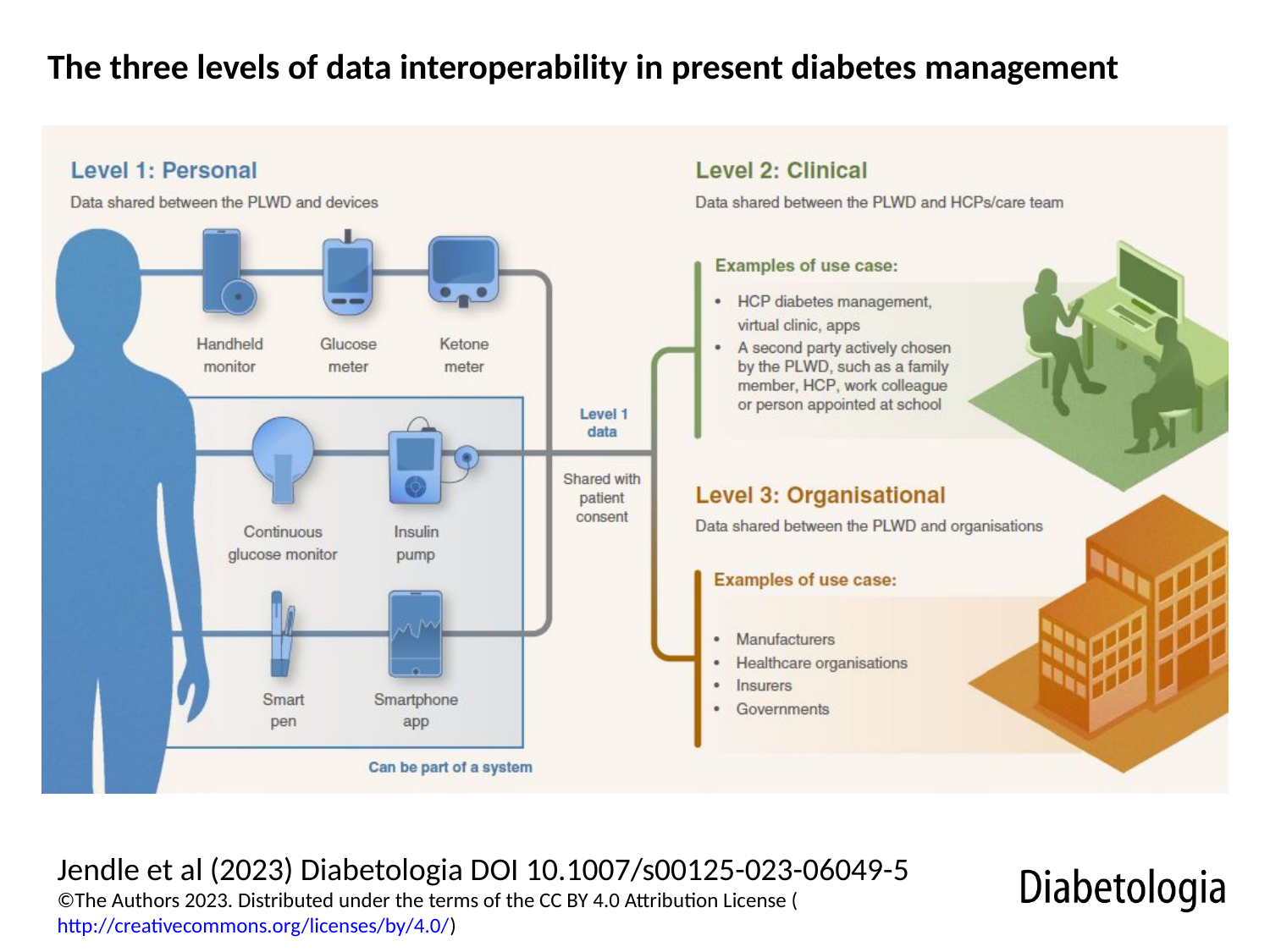

The three levels of data interoperability in present diabetes management
Jendle et al (2023) Diabetologia DOI 10.1007/s00125-023-06049-5
©The Authors 2023. Distributed under the terms of the CC BY 4.0 Attribution License (http://creativecommons.org/licenses/by/4.0/)
